# Visualization of Balbiani Body disassembly during human primordial follicle activation

**DOI:** 10.17912/micropub.biology.000989

**Published:** 2023-10-17

**Authors:** Ruchi Amin, Orhan Bukulmez, Jeffrey B. Woodruff

**Affiliations:** 1 Obstetrics and Gynecology, The University of Texas Southwestern Medical Center, Dallas, Texas, United States; 2 Cell Biology, The University of Texas Southwestern Medical Center, Dallas, Texas, United States

## Abstract

Dormant human oocytes contain a perinuclear super-organelle, called the Balbiani Body, which is not present in mature oocytes. Here, we use confocal imaging to visualize two Balbiani Body markers—mitochondria and the DEAD-box helicase DDX4—in preantral follicles isolated from a 20-year-old female patient. In primordial follicles, mitochondria were concentrated in a ring near the oocyte nucleus, while DDX4 formed adjacent micron-scale spherical condensates. In primary and secondary follicles, the mitochondria were dispersed throughout the oocyte cytoplasm, and large DDX4 condensates were not visible. Our data suggest that the Balbiani Body breaks down during the primordial to primary follicle transition, thus releasing mitochondria and soluble DDX4 protein into the oocyte cytoplasm.

**Figure 1. Subcellular localization of Balbiani Body components in quiescent and activated human oocytes:**
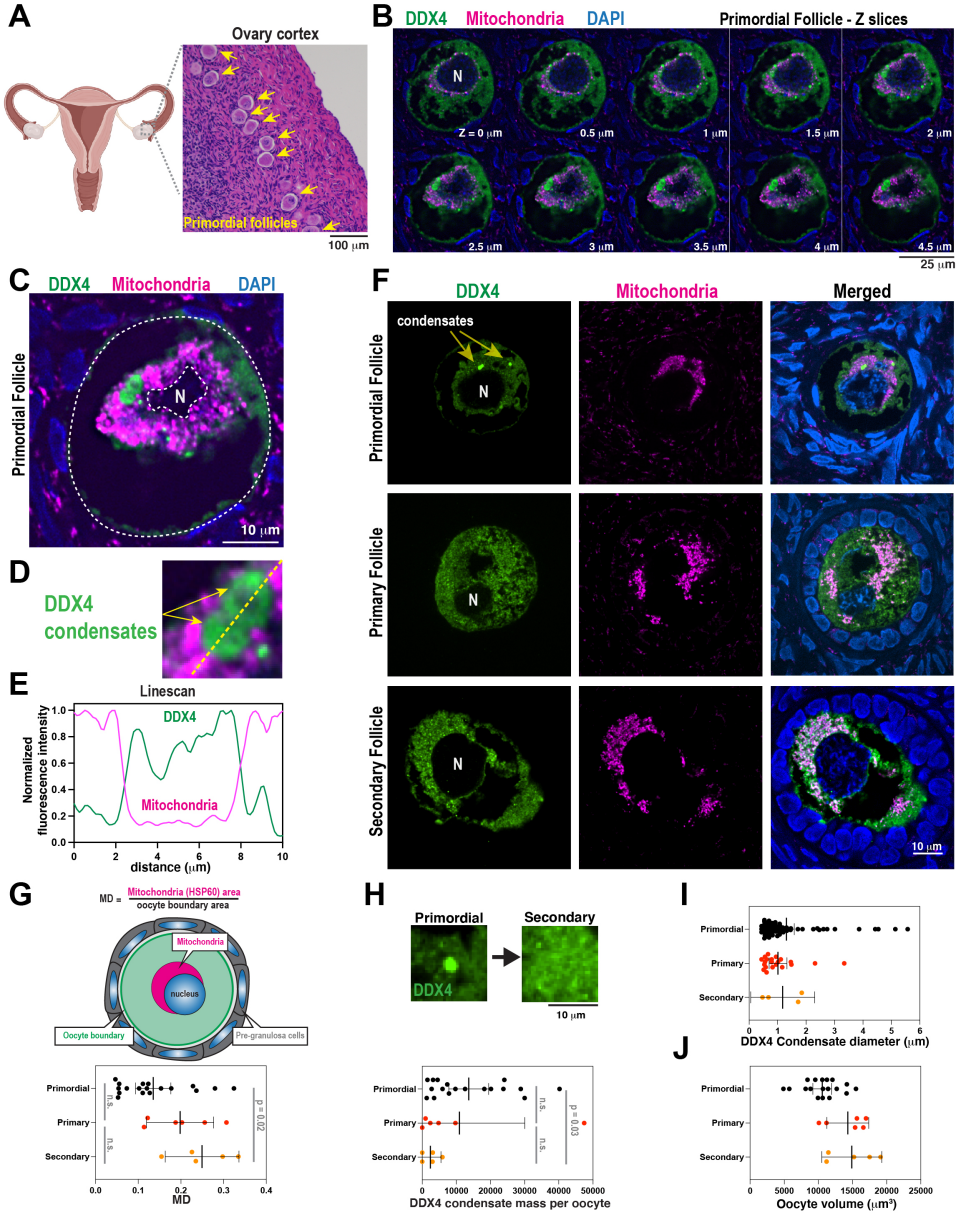
A. Left, quiescent oocytes reside within primordial follicles in the ovarian cortex. Right, hematoxylin and eosin staining of ovarian cortical tissue surgically resected from a 20-year-old patient with no history of infertility. Diagram is modified from images created with BioRender.com. B. Z-slices (0.5 μm in distance) of a primordial follicle. Ovarian tissue was fixed, sectioned, and stained with antibodies against DDX4 (VASA homolog) and HSP60 (mitochondrial protein) and DAPI (DNA). Tissues were mounted on glass slides and imaged with fluorescence confocal microscopy. The nucleus is labeled with “N”. C. Representative slice from B. D. Zoomed-in image from C) highlighting perinuclear accumulations of mitochondria that surround prominent foci of DDX4 called “condensates”. E. Measurement of fluorescent intensity across DDX4 condensates embedded within the perinuclear mitochondrial cloud. Fluorescent intensities were normalized to peak intensities (peak = 1). F. Comparison of DDX4 and HSP60 (Mitochondria) signal in quiescent (primordial stage) and activated oocytes (primary and secondary stage). The nucleus is labeled with “N”. G. Quantification of mitochondrial dispersal per oocyte in different stages of follicle development (mean +/- 95% C.I.; n = 18 primordial; n = 6 primary; n = 5 secondary). Significance was calculated by a Kruskal-Wallis multiple comparisons test. H. Quantification of DDX4 condensate mass per oocyte in different stages of follicle development (mean +/- 95% C.I.; n = 18 primordial; n = 6 primary; n = 5 secondary). Significance was calculated by a Kruskal-Wallis multiple comparisons test. I. Diameters of DDX4 condensates (mean +/- 95% C.I.; n = 67 primordial; n = 21 primary; n = 4 secondary). J. Volumes of oocytes in different stages of follicle development (mean +/- 95% C.I.; n = 18 primordial; n = 6 primary; n = 5 secondary).

## Description

Female humans generate their entire reserve of oocytes during the mid-gestation period and store them until sexual maturity and beyond. During the long storage period, these dormant oocytes remain arrested in the diplotene phase of meiotic prophase I and are surrounded by a single layer of flattened pre-granulosa cells. The oocyte and pre-granulosa cells together comprise the primordial follicle (Li and Albertini, 2013) . Multiple signaling pathways have been implicated in the activation of oocytes and subsequent development of the primary follicle, but the exact mechanism is not known (Lee and Chang, 2019) . How oocytes survive long-term storage is a long-standing open question.

During formation, oocytes undergo dramatic restructuring of their cytoplasm. The oocytes of many egg-storing species package their mitochondria, RNA, Golgi, ribosomes, and various proteins in perinuclear super organelles (Boke et al., 2016; Hertig and Adams, 1967; Jamieson-Lucy and Mullins, 2019; Kloc et al., 2004; Woodruff et al., 2018) . In zebrafish, frogs, and insects, this structure is called a Balbiani Body, and it is built from the prion-like proteins Bucky ball and Xvelo (Boke et al., 2016; Marlow and Mullins, 2008) . These studies demonstrated that the Balbiani Body stores germ plasm needed to direct formation of the future germline. They proposed that the Balbiani Body packages maternal elements needed for oocyte development and early embryogenesis prior to zygotic genome activation (Marlow and Mullins, 2008) . The Balbiani Body stores these contents during oocyte arrest and then releases them after oocyte activation.

Negative stain electron microscopy of human primordial follicles identified a dense collection of mitochondria, other organelles, and membranes adjacent to the oocyte nucleus, which the authors termed a Balbiani Body (Hertig and Adams, 1967) . However, it is not known how functionally similar this structure is to Balbiani Bodies seen in non-mammalian species. During mammalian embryogenesis, specification of the germ lineage is inductive, obviating the need for polarized storage of germ plasm in the oocyte (Seydoux and Braun, 2006) . Furthermore, Bucky ball and Xvelo proteins have no clear homologs in humans. Mouse primary oocytes do not cluster their mitochondria at all and instead form a Golgi-rich ring around the nucleus (Dhandapani et al., 2022) .

Little is known about the architecture, composition, and dynamics of the Balbiani Body in human oocytes. Culture of primordial follicles that recapitulates long-term oocyte dormancy is not currently possible (McLaughlin et al., 2018) . Thus, analyzing human primordial follicles requires surgical removal of ovarian tissue from patients and immediate processing to conserve natural architecture. Immunohistochemical staining of primordial follicles identified DEAD-Box Helicase 4 (DDX4; homolog of VASA) in a perinuclear region of oocytes (Albamonte et al., 2013, Albamonte et al., 2008, Dhandapani et al., 2022) , suggesting that DDX4 is a Balbiani Body component. However, the spatial relationship between DDX4 and other Balbiani Body components, such as mitochondria, has not been examined thoroughly. Thus, in humans, it is not known if the Balbiani Body is a uniform protein phase that surrounds mitochondria or a heterogenous, multi-phase aggregate. Nor is it clear when the Balbiani Body breaks down during oocyte development. Toward clarifying these unknowns, we used fluorescence confocal microscopy and deconvolution to examine preantral oocytes isolated from a living 20-year-old patient with no history of infertility or cancer.

To preserve native cytoplasmic structures within oocytes, we surgically removed strips of the ovarian cortex and immediately fixed the tissue in 4% formaldehyde. Hematoxylin and eosin staining showed oocytes and surrounding somatic cells with the expected morphology, indicating that the tissue was healthy at the time of fixation ( Figure 1A ). We identified primordial follicles based on the presence of a single layer of flattened pre-granulosa cells surrounding an oocyte of ~35 μm diameter (Gougeon, 1996; Westergaard et al., 2007).

We then sectioned the tissue into 5 μm slices and used immunofluorescence to label mitochondria (HSP60 antibody) and DEAD-box Helicase 4 (DDX4 antibody). To examine oocyte substructures at high resolution, we imaged the ovarian sections using a confocal microscope equipped with a 100X silicone immersion objective (1.35 NA). Images were further resolved using deconvolution ( Figure 1B,C ). In oocytes contained in the primordial follicles, we saw that HSP60 and DDX4 staining was concentrated in a crescent-shaped ring around the nucleus, consistent with previous reports (Albamonte et al., 2013; Hertig and Adams, 1967) .

Our imaging revealed concentrations of DDX4 in micron-scale spherical compartments, consistent with a previous study (Dhandapani et al., 2022) . These condensates did not overlap with mitochondria, indicating that they occupy spatially distinct sub-compartments within the Balbiani Body ( Figure 1D,E ). When over-expressed in cells or reconstituted in low salt buffer *in vitro* , DDX4 condenses into spherical droplets via liquid-liquid phase separation (Klosin et al., 2020; Nott et al., 2015) . Our results thus suggest that the Balbiani Body is a heterogeneous amalgam of mitochondria and liquid- or gel-like DDX4 condensates.

We then examined primary and secondary follicles in which oocytes have been activated to exit prolonged arrest. These follicles were staged based on the presence of a single (primary) or double (secondary) layer of cuboidal granulosa cells surrounding the oocyte (Gougeon, 1996). In the oocytes contained in these follicles, mitochondria were no longer concentrated in a perinuclear ring but rather distributed throughout the cytoplasm ( Figure 1F,G ). This result is consistent with mitochondrial dispersal that occurs coincident with Balbiani Body breakdown in Xenopus and Zebrafish oocytes (Gupta et al., 2010; Yang et al., 2022) . The cumulative mass of DDX4 condensates per oocyte was lower in more mature oocytes ( Figure 1H ), and the remaining condensates were smaller and fewer compared to those found in primary follicles ( Figure 1I ). Diffuse DDX4 was still present in the cytoplasm of these maturing oocytes. Our data suggest that Balbiani Body breakdown is defined by dispersal of mitochondria into the cytoplasm and dissolution of DDX4 condensates. We conclude that Balbiani Body breakdown occurs at the primordial to primary follicle transition in human oocytes, and that this timing is conserved across multiple vertebrate clades.

In summary, we used fluorescence confocal microscopy to visualize the subcellular architecture of oocytes from a healthy 20-year-old patient. Human Balbiani Bodies contain micron-scale DDX4-positive condensates that are spatially distinct from the mitochondria. Although it was previously assumed that the Balbiani Body disassembles during oocyte maturation, we show that the mitochondria and DDX4 disperse into oocyte cytoplasm at the primordial-primary follicle transition. We propose that disassembly of the Balbiani Body is triggered by oocyte activation and may be one of the first requirements for oocyte maturation.

DDX4 condensates have been reported to sequester housekeeping mRNA transcripts under stress (Klosin et al., 2020; Nott et al., 2015) and regulate translation of germline-specific transcripts (Raz, 2000) . It is possible that the Balbiani Body functions to trap and maintain DDX4 condensates to store and protect mRNAs needed for oocyte maturation or embryogenesis prior to zygotic genome activation. Disassembly of the Balbiani Body would release these condensates, exposing them to cytoplasmic agents that promote dissolution and release of their mRNA. The volume of the oocyte cytoplasm increases coincident with Balbiani Body breakdown, which could also facilitate condensate dissolution ( Figure 1J ) (Westergaard et al., 2007) . Furthermore, the packaging of mitochondria adjacent the nucleus may act as a protective mechanism to reduce the production of reactive oxygen species (ROS) or select for healthy mitochondria (Jamieson-Lucy and Mullins, 2019) .

Oocytes are some of the longest-lived cells in the mammalian body, surviving up to 50 years in humans. Since the Balbiani Body is unique to dormant oocytes, it could be key to preserving oocyte quality during decades of storage. It is possible that as females age this line of defense does not function properly, resulting in a higher percentage of genetically abnormal oocytes. Our work establishes nanometer-scale morphological metrics for Balbiani Body integrity, which could be a readout of primary oocyte quality. Our results also provide useful benchmarks for improving *in vitro* culture of follicles or vitrification of ovarian strips used for transplantation.

## Methods

**Patient Information**

A 20-year-old female patient with no known pathology or history of infertility elected for total hysterectomy and bilateral salpingo-oopherectomy for purposes of transgender care. Informed consent was received prior to the procedure. Use of human ovarian tissue was approved by the Institute Review Board of the University of Texas Southwestern Medical Center (IRB#0801-404/012012-185).

**Ovarian tissue preparation**

Ovarian tissue, from both ovaries, was placed in 4% formaldehyde directly from the operating room and incubated for 24 hours for fixation. The tissue was embedded in paraffin and systematically cut into 5 μm sections by Leica Rotary Microtomes. Routine hematoxylin-eosin (H&E) staining was performed on tissue slices from each ovary. The remaining sections were allocated for immunohistochemistry. Ovarian follicle identification and categorization on H&E-stained tissue was performed according to Gougeon classification (Gougeon, 1996).

To prepare the tissue slices for immunohistochemistry, the mounted paraffin sections were dewaxed and rehydrated in xylene and serial dilutions of ethanol, respectively. Following a wash step with phosphate-buffered solution (PBS), an antigen retrieval step was performed using a basic retrieval reagent (R&D, CTS013, USA) at 90-95°C for 5 minutes. The slides were cooled completely in PBS prior to processing.

**Immunohistochemistry**

To assess mitochondrial and DDX4 expression and localization, the tissue was inserted into a solution containing 10% goat serum, 3% bovine serum albumin, and 0.03% Triton X-100 (blocking solution) for one hour. The tissue was then incubated overnight with primary antibody at 4°C. The following primary antibodies were used: directly-labeled mouse monoclonal anti-HSP60 (Proteintech, 1:500 dilution; 66041-1-Ig, USA) and rabbit polyclonal anti-DDX4/VASA (Abcam, 1:500 dilution; ab277638, USA). Following primary antibody incubation, a series of washes were performed with PBS. The tissue was then incubated with a goat anti-rabbit secondary antibody (Invitrogen, 1:1000 dilution, A-11008) for 1 hour prior to staining for DNA with Vectashield (DAPI) antifade mounting media (Vectorlabs, H-1000-10, USA). Negative controls were included by incubating with no primary antibody.

The directly labeled anti-HSP60 was created using an Alexa Fluor 647 Lightning-Link kit (Abcam, USA) following their instructions. This antibody was kept in the dark for storage and during incubations.

**Confocal Imaging**

Images were acquired using an inverted Nikon Eclipse Ti2-E microscope with a Yokogawa confocal scanner unit (CSU-W1), piezo Z stage, an iXon Ultra 888 EMCCD camera (Andor), a 100X (1.35 NA) silicone oil objective, 0.5 micron steps, and 1 X 1 binning. Images were deconvolved using the Nikon Elements 3D deconvolution algorithm (22 iterations, type: automatic). Image locations were coded by position on the stage to ensure oocytes were measured only once. Images were analyzed using FIJI ( https://imagej.net/software/fiji/ ). For mitochdondria dispersal, the oocyte cytoplasm was segmented using a thresholding method (green signal, include holes), an ROI generated, then converted to a mask. All HSP60 signal outside the mask was removed. HSP60 signal inside the oocyte was segmented by thresholding and the particle analyzer function, then measured. For condensate dissolution, condensates were identified and measured using thresholding and the particle analyzer function. For each oocyte, the integrated densities of condensates were summed and displayed.

**Statistics and data representation**

Plotting of data and statistical analyses were performed using GraphPad Prism 9. The sample size, relevant statistical test, and significance are included in each figure legend or on the figure itself.
